# HPV genotype distribution in Brazilian women with and without cervical lesions: correlation to cytological data

**DOI:** 10.1186/s12985-016-0594-3

**Published:** 2016-08-12

**Authors:** Toni Ricardo Martins, Cristina Mendes de Oliveira, Luciana Reis Rosa, Cristiane de Campos Centrone, Célia Luiza Regina Rodrigues, Luisa Lina Villa, José Eduardo Levi

**Affiliations:** 1 Institute of Tropical Medicine, Universidade de São Paulo, Virology Laboratory / LIM 52, Dr. Enéas Carvalho de Aguiar, 470, CEP: 05403-000 São Paulo, SP Brazil; 2 Department of Infectious Diseases, Universidade de São Paulo School of Medicine, São Paulo, Brazil; 3 Department of Oncology and Radiology, Universidade de São Paulo, School of Medicine, São Paulo, Brazil; 4 National Institute of Science and Technology of the Diseases Associated to the Papillomaviruses - INCT-HPV, São Paulo, Brazil

**Keywords:** Cervical Cancer, Cytology, HPV, HPV types, PapilloCheck

## Abstract

**Background:**

Human Papillomavirus (HPV) genotype distribution varies according to the method of assessment and population groups. This study analyzed type-specific HPV infections among women ranging from 14–95 years old, displaying normal and abnormal cytology, from São Paulo and Barretos cities, Brazil.

**Methods:**

Women found positive for High Risk-HPVs DNA by either the Hybrid Capture 2 (HC2) or Cobas HPV Test (*n* = 431) plus a random sample of 223 negative by both assays and 11 samples with indeterminate results, totalizing 665 samples, were submitted to HPV detection by the PapilloCheck test. Cytological distribution included 499 women with a cytological result of **N**egative for **I**ntraepithelial **L**esion or **M**alignancy and 166 with some abnormality as follows: 54 **A**typical **S**quamous **C**ells of **U**ndetermined **S**ignificance; 66 **L**ow-Grade **S**quamous **I**ntraepithelial **L**esion; 43 **H**igh-Grade **S**quamous **I**ntraepithelial **L**esion and 3 (0.5 %) **I**nvasive **C**ervical **C**ancer.

**Results:**

From the 323 samples (48.6 %) that had detectable HPV-DNA by the PapilloCheck assay, 31 were HPV negative by the cobas HPV and HC2 assays. Out of these 31 samples, 14 were associated with HR-HPVs types while the remaining 17 harbored exclusively low-risk HPVs. In contrast, 49 samples positive by cobas HPV and HC 2 methods tested negative by the PapilloCheck assay (19.8 %). Overall, the most frequent HR-HPV type was HPV 16 (23.2 %), followed by 56 (21.0 %), 52 (8.7 %) and 31 (7.7 %) and the most frequent LR-HPV type was HPV 42 (12.1 %) followed by 6 (6.2 %). Among the HR-HPV types, HPV 56 and 16 were the most frequent types in NILM, found in 19.1 and 17.7 % of the patients respectively while in HSIL and ICC cases, HPV 16 was the predominant type, detected in 37.2 and 66.7 % of these samples.

**Conclusions:**

In the population studied, HPV 16 and 56 were the most frequently detected HR-HPV types. HPV 56 was found mainly in LSIL and NILM suggesting a low oncogenic potential. HPV 16 continues to be the most prevalent type in high-grade lesions whereas HPV 18 was found in a low frequency both in NILM and abnormal smears. Surveillance of HPV infections by molecular methods is an important tool for the development and improvement of prevention strategies.

## Background

Two hundred different HPV types, classified into 49 species, are currently recognized by the International HPV Reference Center [1]. From these, about 40 are found in the female genital tract [2] and can be divided, according to their ability to generate malignancies, into low-risk types (LR-HPV) (e.g. HPV 6 and 11) which are rarely detected in high grade cervical lesions (HSIL) but produces the majority of genital warts [3, 4] and high-risk types (HR-HPV), which cause lesions with potential for oncogenic progression (e.g. HPV 16 and 18) [5]. Longitudinal studies have shown that individual HPV types grouped under the HR-HPV class differ enormously in their oncogenic potential and the pace in which they may drive the evolution of high-grade premalignant lesions to cervical cancer. Knowledge on the distribution of individual HPV types in different geographical areas is fundamental for the optimization of cervical cancer preventive strategies such as vaccination and HPV-DNA primary screening, which should take into consideration the local contribution of HPV types [6].

The majority of HPV infected women will never develop cancer since most HPV infections are transient and asymptomatic. As expected, HPV is more prevalent in young sexually active women (18 to 30 years) [7], decaying with aging. However, a second peak of HPV has been described after menopause in some studies [8], but was not verified in larger cohorts worldwide, except for Africa and Central America [7]. HPV persistence and progression from infection to disease vary according to the virus genotype, even among HR-HPV types [9]. Another important factor for disease progression seems to be the age; women with an HPV genital infection and older than 40 years have much higher risk of progression to cervical cancer than younger women [10].

Invasive Cervical Cancer (ICC) is worldwide the third most frequent cancer among women [11]. In Brazil, ICC is preceded only by non-melanoma skin cancer and breast cancer. According to the Brazilian National Cancer Institute, 16,340 new cases are expected to occur in 2016 with an estimated incidence of 15.85 cases per 100,000 women. In São Paulo State, where the present study was conducted, the estimated incidence for 2016 was of 9.50 per 100,000 women, in contrast to, for instance, another Brazilian city, Manaus located in the North region, where an alarming projection of 53.73 cases per 100,000 women, not adjusted by age, was made [12].

The distribution and prevalence of the HPV genotypes vary with the grade of cervical disease, age and the geographical location of the patients [8, 13–15]. Prevalence of HPV 16 and 18 increases in parallel to the severity of the lesion [16–18] being associated to approximately 70 % of the ICC cases worldwide but are also the most frequent types observed in women with normal cytology [7]. In a meta-analysis of 1 million women with normal cytological findings, HPV18 was found in many regions as the second most frequent [19], also verified in some Brazilian studies [20–22]. Notwithstanding HPV 31 occupies the second position in frequency in Europe and HPV 52 in Africa, pointing to the importance of other HR-types non 16/18 in certain regions [19]. Illustrating the geographical variability, in Brazil, HPVs 31 and 33 are the second most prevalent types [23, 24] among selected populations, respectively in the Northeast and Central regions. Surprisingly, HPV 66 was detected in 22 % of the HPV positive samples from Campo Grande, Mato Grosso do Sul, [25] and HPV 58 was the most frequent type (19.8 %) in HIV-infected women followed by HPV 53 (15.5 %) in the Southeast region [26].

As a result of accumulated knowledge over the past three decades on the causal relationship between HPV infection and ICC, detection of the viral DNA became an attractive approach to identify women at risk for developing ICC [27, 28]. Evidence from randomized trials now supports the incorporation of screening methods that clearly focus on the detection of the HPV genome [29] since there is overwhelming evidence that this is more sensitive than cytology for identifying CIN3, the true premalignant precursor of ICC [30–32]. Investigating HPV genotypic distribution and disease-association became an important tool in epidemiological studies, further supporting vaccine and screening tests development and improvement, by ensuring their coverage and effectiveness [33, 34].

Since June 2013, the Brazilian Ministry of Health has announced the decision to introduce the quadrivalent HPV vaccine in the national immunization program, the vaccination schedule (0 and 6 months) was updated in January 2016. From 2014, this vaccine was offered to girls and adolescents 11–13 years old and from 2015 for adolescents between 9 and 11 years [35]. In this context, HPV genotyping may be an essential tool to assess the program effectiveness by identification of the genotypic profile pre and post HPV vaccination.

PapilloCheck assay is a robust method for HPV genotyping, which is based on PCR amplification of a fragment of approximately 350 bp from the E1 region of HPV genomes, using broad-spectrum consensus primers, allowing the simultaneous detection and genotyping of 24 different HPV types by DNA chip technology including low-risk HPVs [36].

We aimed to uncover the HPV type-distribution hiding under an HPV-HR result in the HC2 test or the 12 HR-HPVs cocktail in the Cobas HPV test to analyze the HPVs genotype distribution in women undergoing routine cervical cancer screening and women with a previous abnormal Pap test in São Paulo and Barretos cities and their correlation to cytological results and age groups.

## Methods

### Study population

Specimens were collected in between October, 2009 and April, 2011, in two reference centers of São Paulo State, Brazil: Hospital das Clínicas from the São Paulo Medical School, Universidade de São Paulo and Hospital de Câncer de Barretos. Participants, with age ranging from 14 to 95 years, average 43 yo, median 43yo (SD = ±14 yo), consisted of 403 women undergoing routine screening (*n* = 156 from São Paulo and 247 from Barretos), in addition to 262 referral women with a previous abnormal Pap test in the year preceding study enrollment (163 from São Paulo and 99 from Barretos). A cervical sample was obtained and processed as described elsewhere [14].

Briefly, all samples were submitted to Cobas 4800 HPV (Roche Molecular Systems, Pleasanton, CA, USA) and the Hybrid Capture 2 (HC2, Qiagen, Hilden, Germany) assays. Only the HR-HPV cocktail probe was employed on the HC2 testing. Samples from women found positive for high-risk HPVs DNA by either the HC2 or Cobas HPV (*n* = 431) were submitted to HPV genotyping by the PapilloCheck assay (Greiner Bio-One, Frickenhausen, Germany) [14] plus a random sample of 223 negative by both assays and 11 samples with indeterminate or invalid results, totalizing 665 samples as shown in Table 1.

**Table 1:** Distribution of samples according to prior molecular testing results

HC2				
Cobas 4800	Negative	Positive	Indet/Inv^a^	Total
	n	n	n	n
Negative	223	68	-	291
Positive	116	247	-	363
Indet/Inv^a^	-	-	11	11
Total	339	315	11	665

Legend: ^a^Indet/Inv: Indeterminate or invalid result

### Molecular tests

Cervical scrapes obtained by the SurePath collection kit (BD SurePath ™ - TriPath, Burlington, NC, EUA) and preserved in liquid cytology medium were transported to the lab where DNA was extracted using the QIAamp DNA Mini Kit (Qiagen, Gaithersburg, USA), according to manufacturer’s instructions. For each reaction, 5 μL of DNA eluate was used in the PapilloCheck assay. Specimens containing the target DNA are hybridized to specific oligonucleotide probes immobilized on a DNA chip and detected by the binding of a Cy5-dUTP labeled oligonucleotide probe to the tag sequence. The DNA chip is scanned by the CheckScanner apparatus at wavelengths of 532 and 635 nm. This test detects HPV genotypes 6, 11, 16, 18, 31, 33, 35, 39, 40, 42, 43, 44, 45, 51, 52, 53.56, 58, 59, 66, 68, 70, 73 and 82. In addition, human ADAT1 gene (adenosine deaminase, tRNA specific 1) is used as an internal control to assess the quality of the DNA [36].

### Cytology

Six hundred sixty five **s**mears were examined by staff cytopathologists at both institutions and classified according to the Bethesda system [37]: NILM (Negative for Intraepithelial Lesion or Malignancy), ASC-US (Atypical Squamous Cells of Undetermined Significance), ASC-H (Atypical squamous cells – cannot exclude HSIL), LSIL (Low-Grade Squamous Intraepithelial Lesion); HSIL (High-Grade Squamous Intraepithelial Lesion) and ICC (Invasive Cervical Cancer). For analysis purposes in this study ASCUS and ASC-H were grouped under ASCUS class.

### Statistical analyses

HPV genotype distribution analyses were performed using GraphPad Prism version 5.0. The *x*^*2*^ and p trend test were used to evaluate statistical significance between genotype, cytological diagnosis and age. For all analyses, a *p* < 0.05 was considered statistically significant.

## Results

### Liquid based cytology

Cytological diagnosis of these samples included 499 (75 %) women with negative cytology (NILM) and 166 (25.0 %) with some cytological abnormality as follows: 54 (8.1 %) ASCUS; 66 (9.9 %) LSIL; 43 (6.5 %) HSIL and 3 (0.5 %) ICC.

When stratified by age group, LSIL was less frequent in women older than 45 years old (p trend = 0.01), whereas HSIL was more frequent (p trend = 0.03). All 3 ICC cases were found in women older than 45 years: 48, 52 and 84 years old.

### HPV genotype distribution

Among the 665 samples analyzed 323 (48.6 %) were HPV positive by the PapilloCheck assay. Single type infection was observed in 65 % (210/323) of them, while 59 (18 %) had co-infection by 2 HPV types, 34 (11 %) by three, 14 (4 %) by four and 6 (2 %) by five or more types. The frequency of HPV infection decreased with aging (p trend <0.001); in women under 31 years old the prevalence of HPV DNA was 58.2 % (92/158) decreasing to 52.3 % (116/222) in women between 31 and 45 years and 40.4 % (115/285) in women older than 45 years old.

The most frequent HR-HPV type among HPV positive women was 16 (23.2 %), followed by 56 (21.0 %), 52 (8.7 %), 31 (7.7 %), 53 (7.7 %), 51 (7.4 %), 39, 59 and 66 (6.5 % each), 33 (5.3 %), 58 (5 %), 18 (5 %), 82 (4 %), 45 and 70 (4 %) each, 68 (3.4 %), 73 (2.5 %) and 35 (2.5 %). The frequency of LR- HPV types was: 42 (12.1 %), 6 (6.2 %), 44 (4.3 %), 43 (4 %), 40 (2.8 %) and 11 (1.5 %). No association between LR-HPV and HR-HPV types with age group was observed.

When stratified by women undergoing routine screening and those with a previous abnormal Pap test (referral), HPV 16 was detected more often in referral than in the screening group; 14.9 % *vs* 8.9 %. This was also observed for HPV 56, the second most common genotype in both groups, found in 13.7 and 7.9 % respectively. Table 2 shows the distribution of the more frequent HR-HPV genotypes in both groups.

**Table 2:** Frequency of HR-HPVs in screening (*n* = 403) and referral (*n* = 262) groups as determined by the Papillocheck assay

HPV type referral	n (%)	HPV type screening	n (%)
16	39 (14.9)	16	36 (8.9)
56	36 (13.7)	56	32 (7.9)
31	17 (6.5)	52	18 (4.5)
39	13 (5.0)	53	15 (3.7)
66	13 (5.0)	59	12 (3.0)
58	13 (5.0)	51	11 (2.7)
51	12 (4.6)	33	9 (3.4)
52	10 (3.8)	45	9 (3.4)
53	10 (3.8)	31	8 (2.0)
82	10 (3.8)	18	8 (2.0)
18	9 (3.4)	39	7 (1.7)
59	9 (3.4)	66	7 (1.7)
Other	88 (33.6)	Other	67 (16.6)

Comparing the HPV result obtained by the PapilloCheck to the cytology diagnosis, we found 41.8 % (209/499) of HPV positive results among women with negative cytology; 55.6 % (30/54) ASCUS; 80.3 % (53/66) LSIL; 67.4 % (29/43) HSIL and 100 % (3/3) among women with ICC. Eight HSIL cases, 18.6 % (8/43) were positive either by Hybrid Capture 2 (HC2), Cobas HPV or both, while negative for the Papillocheck assay, whereas in the three ICC cases HPV was detected by all three methods.

**Fig. 1:**
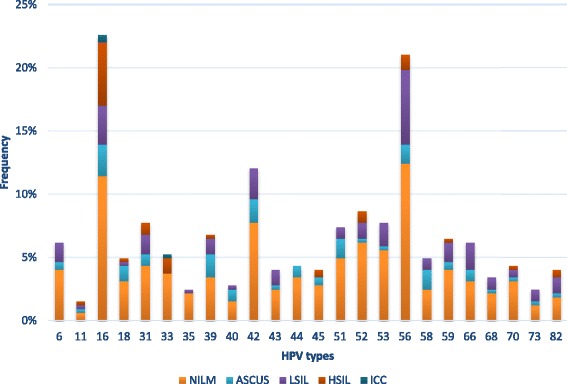
Frequency of individual HPV types according to cytological diagnosis

HPV 56 and HPV 16 were the most frequent in negative cytology being found in 19.1 % (40/209) and 17.7 % (37/209) of the patients, respectively. In LSIL cases, HPV 56 was the most frequent type present in 28.8 % (19/66), while in HSIL and ICC cases, HPV 16 was the predominant type, detected in 37.2 % (16/43) and 66.7 % (2/3) of these samples, respectively whilst, the third ICC case harbored HPV 33. Distribution of HPV types according to the cytological diagnosis is shown in Fig. 1.

Concerning samples that showed negative results in molecular screening by HC2 and Cobas HPV on the original study, 13.9 % (31/223) were HPV positive by the Papillocheck. Of these, 54.8 % (17/31) were positive for LR-HPV, whereas 45.2 % (14/31) were associated with HR- HPVs types 16, 33, 39, 51, 53, 56, 58, 59, 66, 68, 70 or 73. In contrast, 49 samples positive by both Cobas HPV and HC 2 methods tested negative by the PapilloCheck assay (19.8 %; 49/247).

**Fig. 2:**
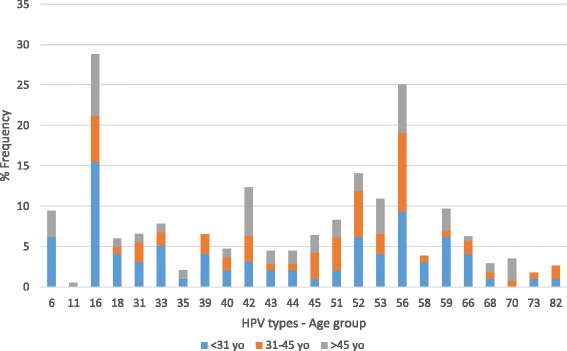
Distribution of HPV genotypes by age (screening group, *n* = 403)

The two HR-HPV types found more frequently in all ages from the screening group were HPV 16 and 56. HPV 16 was observed in 15.5 % (15/97) of samples from women under 31 years and in 7.7 % (14/183) of samples from women older than 45 years, followed by HPV-56 that was detected in 9.3 % (9/97) of the patients under 31 years and in 6.0 % (11/183) of the older than 45 years. In samples from women aged 31–45 years old, the most frequent HR-HPV type was HPV 56 (9.8 %; 12/123), followed by HPV 16 (5.7 %; 12/123). The most frequent HPV types according to age groups are depicted in Fig. 2.

**Fig. 3:**
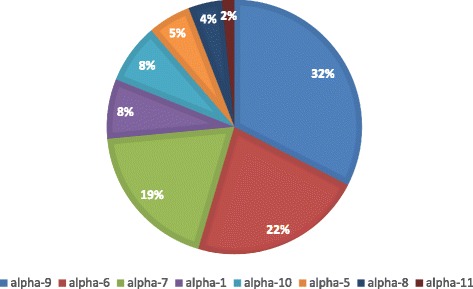
Frequency of HPV genotypes among HPV+ samples as grouped by phylogenetic species

When HR-HPV genotypes are clustered in HPV species according to the classification of de Villiers et al. [38], it may be observed that the most common species were alpha-9 and alpha-6, both composed exclusively of high-risk HPVs; (16, 31, 33, 35, 52, 58) and (53, 56, 66) respectively, followed by species alpha-7, alpha-1, alpha-10, alpha-5, alpha-8 and alpha-11 (Fig. 3).

## Discussion

In the present study, the specimens analyzed were enriched from a group of HPV positive samples and a random subsample of specimens displaying HPV negative molecular testing results, as described above. Thus, it has to be considered that this rate of HPV infection does not represent the true prevalence in women submitted to cervical cancer screening, in whom HR-HPV DNA was detected in about 9 % of subjects under routine screening, nor the prevalence in the referral group, of 24 % in women with a previous abnormal Pap test [14].

Differences in the prevalence and distribution of HPV types worldwide have been observed in several studies [1, 39], and can be related to the complex interaction between distinct HPV types with host immunogenetic factors, age, geographic location of the patients or use of different sets of primers in HPV PCR and genotyping assays used in these studies [40, 41].

HPV 16 was the most frequent type in the present series as found in almost all screening population studies, irrespective of the study design [42–44]. An interesting finding of this study was that HPV 56 was the second most common type, as previously reported by Levi and colleagues [14], confirming the results of Bruno et al. [15] in a study conducted in women from the state of Bahia, Brazil, where it was detected in 14 % of the cervical samples. This finding was not verified in other studies performed in Brazil; therefore, presence of HPV 56 in such a high frequency demands further investigation.

In the current study, HPV 56 was the most frequent type among HPV+ women bearing NILM and LSIL cytology, found respectively in 19.1 and 28.8 % of these samples. In contrast Oliveira-Silva et al. [22] studied 84 NILM samples from Brazilian women reactive by Hybrid Capture II, observing only one HPV 56 infection (0.8 %) while Martin et al. [45], in Spain, reported the presence of HPV 56 in 2.4 and 7.9 % of their NILM and LSIL cytology specimens.

In fact, other studies using PapilloCheck assay reported rates of HPV 56 above the expectation [15, 46], which may be attributed to an increased sensitivity of the test for this particular type or, alternatively, reduced specificity [36]. Undertaking other molecular techniques such as type-specific PCR or DNA direct sequencing of these samples could dismiss the potential lack of specificity of this method.

In this study, HPV 16 was the predominant type found in 38.1 and 66.7 % of the HPV+ HSIL and ICC cases respectively. These findings corroborate the results of a systematic review and meta-analysis in Latin America and the Caribbean including 2446 cases of HSIL and 5540 of ICC that reported HPV 16 as the most frequent type, verified in 46.5 % of HSILs and 53.2 % of ICC cases [47]. In another study conducted in São Paulo, samples from histologically confirmed invasive cervical cancer (*n* = 175) also harbored HPV 16 as the most frequent type (77.6 %), followed by HPV 18 (12.3 %), HPV 31 (8.8 %), HPV 33 (7.1 %) and HPV 35 (5.9 %) [48].

Detection of of LR-HPV types in the current study is explained by the inclusion of probes for these genotypes in the PapilloCheck assay, depicting them when present in co-infections with the HR-HPV genotypes, or in single type infections in the HPV screening (Cobas-/HC2-) negative group. Regarding the LR-HPV types, HPV 42 was the most common as observed in other studies using the same method of genotyping [15, 49, 50]. This higher frequency of HPV 42 compared to other low risk types could potentially be attributed to specific features of the Papillocheck assay, as observed for HPV 56.

Currently, HPV DNA testing has been incorporated into cervical cancer screening algorithms to triage cases with indeterminate cytologic results (ASC-US) [51]; clinical follow-up after treatment, monitoring of patients without cytological lesions or only minor lesions that were identified by colposcopy; resolution of discordant results of cytology, colposcopy, or histology [52, 53]; or as an stand-alone primary screening tool [54, 55]. As seen in this study, HPV DNA is detected in the majority of the precursor lesions or ICC [56]. Several HPV tests are available in the market, and we evaluated methodologies with different targets and principles, nevertheless no test is infallible and false-negative results may possibly occur [57].

In the current study, HPV DNA was not detected in 32.6 % of HSIL samples. This rate is higher than found by Guo et al. [58], whom reported that 13.6 % of their HSIL cases displayed a negative HPV-DNA molecular test. False-negative results may be due to a low burden of HPV DNA, presence of a new type of HPV not targeted by the method used, inadequate sampling, cytological and histopathological missclassification or might have been the result of methodological or reproducibility errors [59, 60]. The fact that the E1 region, targeted by the Papillocheck assay, is located next to the E2 region in the HPV genome, could render it more vulnerable to partial deletion, that often occurs during the HPV DNA integration into the host cell DNA [36] verified on more advanced squamous intraepithelial lesions.

When stratified by age group, women under 31 years old had the highest HPV frequency which mostly represents transient infections [61]. However, for women aged 31 years old or more, test positivity may indicate persistent infection, which would require more intense monitoring, when molecular testing could be more effective.

Multiple infections were detected in 35 % of samples, and were more frequent in women under 31 years old. This observation is consistent with others studies [45, 62], and suggests that it may be associated to more intense sexual activity in younger women. It is well known that the prevalence of HPV infection and the occurrence of multiple infections decrease with an increase in age [63, 64].

Current US guidelines recommend genotyping only for HPV 16 and 18, and detection of either of these two HPV types is sufficient to warrant direct referral to colposcopy, avoiding additional triage methods. However, some studies suggested that it may be also clinically useful to identify other HPV types such as HPV 31, 33, 33/58 (combined), which showed comparable or increased risk for the development of CIN 2+ [65–67].

## Conclusions

The nonavalent HPV vaccine appears to be safe and effective in preventing persistent infection and precancerous lesions associated with HPV types 16/18/31/33/45/52/58, as well as genital warts related to HPV types 6 and 11 [68]. Moreover, the currently available HPV vaccines have shown partial cross-protection against HR-HPV types [69] by alpha7/alpha9 high-risk HPV species but it should not provide cross-protection against HPV 56, which is an alpha 6 group species. Although HPV 56 was a common finding in our study, especially in cases of NILM and LSIL, it was not found in ICC and in HSIL cases suggesting lower oncogenic potential, corroborating the data from de Oliveira et al. [48], who did not find HPV 56 in any of the 175 ICC cases from São Paulo.

The prevalence and genotype distribution of human papillomavirus (HPV) provides the basis for designing HPV prevention programs. HPV types 53, 66, 68, 70, 73 and 82 included in the Papillocheck assay and other types such as HPVs 26 and 67 have been rarely but consistently identified as single HPV infections in about 3 % of cervical cancer tissues [70]. To describe the genotypic profile pre and post HPV immunization is an important step in prevention strategies for cervical cancer and further studies are needed to elucidate the potential role of these HPV types in the development of malignancies, eventually justifing their inclusion in new HPV vaccine formulations. Such decisions have to include careful estimation of effectiveness and cost-benefit.

## References

[1] Kocjan BJ, Bzhalava D, Forslund O, Dillner J, Poljak M (2015). Molecular methods for identification and characterization of novel papillomaviruses. Clin Microbiol Infect.

[2] Munoz N, Bosch FX, de Sanjose S, Herrero R, Castellsague X, Shah KV (2003). Epidemiologic classification of human papillomavirus types associated with cervical cancer. N Engl J Med.

[3] Piana A, Sotgiu G, Castiglia P, Pischedda S, Cocuzza C, Capobianco G (2011). Prevalence and type distribution of human papillomavirus infection in women from North Sardinia, Italy. BMC Public Health.

[4] de Oliveira CM, Aguiar LS, Genta ML, Alves VA, Levi JE (2011). HPV-11 associated metastatic cervical cancer. Gynecol Oncol Case Rep.

[5] Bouvard V, Baan R, Straif K, Grosse Y, Secretan B, El Ghissassi F (2009). A review of human carcinogens--Part B: biological agents. Lancet Oncol.

[6] de Sanjose S, Quint WG, Alemany L, Geraets DT, Klaustermeier JE, Lloveras B (2010). Human papillomavirus genotype attribution in invasive cervical cancer: a retrospective cross-sectional worldwide study. Lancet Oncol.

[7] de Sanjose S, Diaz M, Castellsague X, Clifford G, Bruni L, Munoz N (2007). Worldwide prevalence and genotype distribution of cervical human papillomavirus DNA in women with normal cytology: a meta-analysis. Lancet Infect Dis.

[8] Fernandes JV, Meissner Rde V, de Carvalho MG, Fernandes TA, de Azevedo PR, Villa LL (2009). Prevalence of HPV infection by cervical cytologic status in Brazil. Int J Gynaecol Obstet.

[9] Matsumoto K, Oki A, Furuta R, Maeda H, Yasugi T, Takatsuka N (2011). Predicting the progression of cervical precursor lesions by human papillomavirus genotyping: a prospective cohort study. Int J Cancer.

[10] Powell NG, Hibbitts SJ, Boyde AM, Newcombe RG, Tristram AJ, Fiander AN (2011). The risk of cervical cancer associated with specific types of human papillomavirus: a case–control study in a UK population. Int J Cancer.

[11] WHO. The World Health Organization - Information Centre on HPV and Cervical Cancer (HPV Information Centre). Human Papillomavirus and Related Diseases. 2014. [cited 2014 Out 28] Avaiable at http://www.who.int/hpvcentre. 2014.

[12] INCA. Instituto Nacional do Câncer [Internet]. Ministério da Saúde. [cited 2016 Jan 26]. Available at http://www.inca.gov.br 2016.

[13] Otero-Motta AP, Ordonez JL, Gonzalez-Celador R, Rivas B, Macias Mdel C, Bullon A (2011). Prevalence of human papillomavirus genotypes in cytologic abnormalities from unvaccinated women living in north-western Spain. APMIS.

[14] Levi JE, Longatto-filho A, Eluf-neto J, Rodrigues CL, Oliveira CM, Carloni AC, Lorenzi AT, Tacla M, Fregnani JHTG, Ab’saber AM, Scapulatempo C, Villa LL (2014). Evaluation of HPV molecular tests in primary screening for cervical cancer in Brazil. Open J Obstet Gynecol.

[15] Bruno A, Serravalle K, Travassos AG, Lima BGC (2014). Distribuição dos Genótipos de papillomavírus humano em mulheres do estado da Bahia, Brasil. Rev Bras Ginecol Obstet.

[16] Herrero R, Castle PE, Schiffman M, Bratti MC, Hildesheim A, Morales J (2005). Epidemiologic profile of type-specific human papillomavirus infection and cervical neoplasia in Guanacaste, Costa Rica. J Infect Dis.

[17] Kjaer SK, Breugelmans G, Munk C, Junge J, Watson M, Iftner T (2008). Population-based prevalence, type- and age-specific distribution of HPV in women before introduction of an HPV-vaccination program in Denmark. Int J Cancer.

[18] Li N, Franceschi S, Howell-Jones R, Snijders PJ, Clifford GM (2011). Human papillomavirus type distribution in 30,848 invasive cervical cancers worldwide: Variation by geographical region, histological type and year of publication. Int J Cancer.

[19] Bruni L, Diaz M, Castellsague X, Ferrer E, Bosch FX, de Sanjose S (2010). Cervical human papillomavirus prevalence in 5 continents: meta-analysis of 1 million women with normal cytological findings. J Infect Dis.

[20] Noronha V, Mello W, Villa L, Brito A, Macedo R, Bisi F (1999). Human papillomavirus associated with uterine cervix lesions. Rev Soc Bras Med Trop.

[21] Eluf-Neto J, Booth M, Munoz N, Bosch FX, Meijer CJ, Walboomers JM (1994). Human papillomavirus and invasive cervical cancer in Brazil. Br J Cancer.

[22] Oliveira-Silva M, Lordello CX, Zardo LM, Bonvicino CR, Moreira MA (2011). Human Papillomavirus in Brazilian women with and without cervical lesions. Virol J.

[23] Baldez da Silva MF, Chagas BS, Guimaraes V, Katz LM, Felix PM, Miranda PM (2009). HPV31 and HPV33 incidence in cervical samples from women in Recife, Brazil. Genet Mol Res.

[24] Rabelo-Santos SH, Zeferino L, Villa LL, Sobrinho JP, Amaral RG, Magalhaes AV (2003). Human papillomavirus prevalence among women with cervical intraepithelial neoplasia III and invasive cervical cancer from Goiania, Brazil. Mem Inst Oswaldo Cruz.

[25] Tozetti IA, Scapulatempo IDL, Kawski VL, Ferreira AW, Levi JE (2006). Multiple types of human papillomavirus in cervical samples in women in Campo Grande, MS, Brazil. BJID.

[26] Castilho JL, Levi JE, Luz PM, Cambou MC, Vanni T, de Andrade A (2015). A cross-sectional study of high-risk human papillomavirus clustering and cervical outcomes in HIV-infected women in Rio de Janeiro, Brazil. BMC Cancer.

[27] Cuzick J (1999). Screening for cancer: future potential. Eur J Cancer.

[28] Brismar-Wendel S, Froberg M, Hjerpe A, Andersson S, Johansson B (2009). Age-specific prevalence of HPV genotypes in cervical cytology samples with equivocal or low-grade lesions. Br J Cancer.

[29] Arbyn M, Verdoodt F, Snijders PJ, Verhoef VM, Suonio E, Dillner L (2014). Accuracy of human papillomavirus testing on self-collected versus clinician-collected samples: a meta-analysis. Lancet Oncol.

[30] Mayrand MH, Duarte-Franco E, Rodrigues I, Walter SD, Hanley J, Ferenczy A (2007). Human papillomavirus DNA versus Papanicolaou screening tests for cervical cancer. N Engl J Med.

[31] Sankaranarayanan R, Nene BM, Shastri SS, Jayant K, Muwonge R, Budukh AM (2009). HPV screening for cervical cancer in rural India. N Engl J Med.

[32] Ronco G, Giorgi-Rossi P, Carozzi F, Confortini M, Dalla Palma P, Del Mistro A (2010). Efficacy of human papillomavirus testing for the detection of invasive cervical cancers and cervical intraepithelial neoplasia: a randomised controlled trial. Lancet Oncol.

[33] Sandri MT, Riggio D, Salvatici M, Passerini R, Zorzino L, Boveri S (2009). Typing of human papillomavirus in women with cervical lesions: prevalence and distribution of different genotypes. J Med Virol.

[34] Vidal AC, Murphy SK, Hernandez BY, Vasquez B, Bartlett JA, Oneko O (2011). Distribution of HPV genotypes in cervical intraepithelial lesions and cervical cancer in Tanzanian women. Infect Agent Cancer.

[35] Portal Brasil [Internet] [cited 2016 Jan 26]. Avaiable at http://www.brasil.gov.br/saude/2016/01/calendario-de-vacinacao-atualizado-ja-esta-em-vigor. Accessed 8 Aug 2016.

[36] Dalstein V, Merlin S, Bali C, Saunier M, Dachez R, Ronsin C (2009). Analytical evaluation of the PapilloCheck test, a new commercial DNA chip for detection and genotyping of human papillomavirus. J Virol Methods.

[37] Nayar R, Wilbur DC (2015). The Pap test and Bethesda 2014. Cancer Cytopathol.

[38] de Villiers EM, Fauquet C, Broker TR, Bernard HU, zur Hausen H (2004). Classification of papillomaviruses. Virology.

[39] Capra G, Giovannelli L, Bellavia C, Migliore MC, Caleca MP, Perino A (2008). HPV genotype prevalence in cytologically abnormal cervical samples from women living in south Italy. Virus Res.

[40] Bosch FX, Lorincz A, Munoz N, Meijer CJ, Shah KV (2002). The causal relation between human papillomavirus and cervical cancer. J Clin Pathol.

[41] Trottier H, Franco EL (2006). The epidemiology of genital human papillomavirus infection. Vaccine.

[42] Bosch FX, Burchell AN, Schiffman M, Giuliano AR, de Sanjose S, Bruni L (2008). Epidemiology and natural history of human papillomavirus infections and type-specific implications in cervical neoplasia. Vaccine.

[43] de Ona M, Alvarez-Arguelles ME, Torrents M, Villa L, Rodriguez-Feijoo A, Palacio A (2010). Prevalence, evolution, and features of infection with human papillomavirus: a 15-year longitudinal study of routine screening of a women population in the north of Spain. J Med Virol.

[44] Delgado D, Marin JM, de Diego J, Guerra S, Gonzalez B, Barrios JL (2012). Human papillomavirus (HPV) genotype distribution in women with abnormal cervical cytology in the Basque Country, Spain. Enferm Infecc Microbiol Clin.

[45] Martin P, Kilany L, Garcia D, Lopez-Garcia AM, Martin-Azana MJ, Abraira V (2011). Human papillomavirus genotype distribution in Madrid and correlation with cytological data. BMC Infect Dis.

[46] Schopp B, Holz B, Zago M, Stubenrauch F, Petry KU, Kjaer SK (2010). Evaluation of the performance of the novel PapilloCheck HPV genotyping test by comparison with two other genotyping systems and the HC2 test. J Med Virol.

[47] Ciapponi A, Bardach A, Glujovsky D, Gibbons L, Picconi MA (2011). Type-specific HPV prevalence in cervical cancer and high-grade lesions in Latin America and the Caribbean: systematic review and meta-analysis. PLoS One.

[48] de Oliveira CM, Fregnani JH, Carvalho JP, Longatto-Filho A, Levi JE (2013). Human papillomavirus genotypes distribution in 175 invasive cervical cancer cases from Brazil. BMC Cancer.

[49] Argyri E, Papaspyridakos S, Tsimplaki E, Michala L, Myriokefalitaki E, Papassideri I (2013). A cross sectional study of HPV type prevalence according to age and cytology. BMC Infect Dis.

[50] Vieira L, Almeida A (2013). The cytology and DNA detection by the PapilloCheck((R)) test in the diagnosis of human papillomavirus infection. Eur J Microbiol Immunol (Bp).

[51] Stoler MH, Castle PE, Solomon D, Schiffman M (2007). The expanded use of HPV testing in gynecologic practice per ASCCP-guided management requires the use of well-validated assays. Am J Clin Pathol.

[52] Solomon D (2003). Chapter 14: role of triage testing in cervical cancer screening. J Natl Cancer Inst Monogr.

[53] Schiffman M, Wentzensen N, Wacholder S, Kinney W, Gage JC, Castle PE (2011). Human papillomavirus testing in the prevention of cervical cancer. J Natl Cancer Inst.

[54] Cuzick J, Mayrand MH, Ronco G, Snijders P, Wardle J (2006). Chapter 10: new dimensions in cervical cancer screening. Vaccine.

[55] Ferreccio C, Barriga MI, Lagos M, Ibanez C, Poggi H, Gonzalez F (2013). Screening trial of human papillomavirus for early detection of cervical cancer in Santiago. Chile Int J Cancer.

[56] Castellsague X (2008). Natural history and epidemiology of HPV infection and cervical cancer. Gynecol Oncol.

[57] Castle PE, Solomon D, Wheeler CM, Gravitt PE, Wacholder S, Schiffman M (2008). Human papillomavirus genotype specificity of hybrid capture 2. J Clin Microbiol.

[58] Guo M, Hu L, Baliga M, He Z, Hughson MD (2004). The predictive value of p16(INK4a) and hybrid capture 2 human papillomavirus testing for high-grade cervical intraepithelial neoplasia. Am J Clin Pathol.

[59] Herrington CS (1999). Do HPV-negative cervical carcinomas exist?--revisited. J Pathol.

[60] Peevor R, Bowden S, Jones J, Fiander AN, Hibbitts S (2009). Human Papillomavirus negative but dyskaryotic cervical cytology: re-analysis of molecular testing. J Clin Virol.

[61] Gray SH, Walzer TB (2004). New strategies for cervical cancer screening in adolescents. Curr Opin Pediatr.

[62] Mejlhede N, Bonde J, Fomsgaard A (2009). High frequency of multiple HPV types in cervical specimens from Danish women. APMIS.

[63] Gonzalez-Bosquet E, Esteva C, Munoz-Almagro C, Ferrer P, Perez M, Lailla JM (2008). Identification of vaccine human papillomavirus genotypes in squamous intraepithelial lesions (CIN2-3). Gynecol Oncol.

[64] Sjoeborg KD, Trope A, Lie AK, Jonassen CM, Steinbakk M, Hansen M (2010). HPV genotype distribution according to severity of cervical neoplasia. Gynecol Oncol.

[65] Wright TC, Stoler MH, Behrens CM, Apple R, Derion T, Wright TL (2012). The ATHENA human papillomavirus study: design, methods, and baseline results. Am J Obstet Gynecol.

[66] Halfon P, Lindemann ML, Raimondo A, Ravet S, Camus C, Khiri H (2013). HPV genotype distribution according to severity of cervical neoplasia using the Digene HPV genotyping LQ test. Arch Virol.

[67] Wheeler CM, Hunt WC, Cuzick J, Langsfeld E, Pearse A, Montoya GD (2013). A population-based study of human papillomavirus genotype prevalence in the United States: baseline measures prior to mass human papillomavirus vaccination. Int J Cancer.

[68] Chatterjee A (2014). The next generation of HPV vaccines: nonavalent vaccine V503 on the horizon. Expert Rev Vaccines.

[69] Malagon T, Drolet M, Boily MC, Franco EL, Jit M, Brisson J (2012). Cross-protective efficacy of two human papillomavirus vaccines: a systematic review and meta-analysis. Lancet Infect Dis.

[70] Halec G, Alemany L, Lloveras B, Schmitt M, Alejo M, Bosch FX (2014). Pathogenic role of the eight probably/possibly carcinogenic HPV types 26, 53, 66, 67, 68, 70, 73 and 82 in cervical cancer. J Pathol.

